# SUCCESSFUL CARE CONFERENCES: NURSING HOME STAFF, PERSONS WITH DEMENTIA AND CAREGIVER PERSPECTIVES

**DOI:** 10.1093/geroni/igad104.3608

**Published:** 2023-12-21

**Authors:** Gail Towsley, Linda Edelman, Shelby Montez

**Affiliations:** University of Utah, Salt Lake City, Utah, United States; University of Utah, Salt Lake City, Utah, United States; University of Utah, Salt Lake City, Utah, United States

## Abstract

Work demands of the care team, time constraints, and a lack of guidance from the regulatory environment often result in short, perfunctory mandated quarterly care conferences (e.g., care plan meetings) that lack focus on resident preferences. Me & My Wishes is a video recording approach for documenting, viewing, listening, and discussing nursing home (NH) residents’ living with dementia preferences for daily and end-of-life in care conferences. As part of a study adapting Me & My Wishes for remote delivery, we used a qualitative descriptive design to conduct focus groups (February – June 2023) with experts representing persons living with dementia (PLWD) (n=6), informal care partners (CPs) (n=8), and NH staff (n=9) to inform us of what comprises a successful care conference. We coded and analyzed audio-recorded transcripts, resolved coding discrepancies in team meetings, and derived domains from finalized codes. All groups discussed three primary domains characterizing a successful care conference: 1) resident- engagement; 2) key staff and CPs identified and engaged to support the resident perspective; and 3) structured meetings led by trained facilitators for resident-centered discussion and problem-solving. NH Experts noted that a facilitator who anticipates and mitigates potential barriers (e.g., conflicting discourse) is key for a successful care plan meeting. PLWD identified providing technology assistance for the resident and CPs as important. Care conferences that allow virtual attendance of staff and CPs who know the resident well, are resident-driven, and are well organized can facilitate communication and problem solving resulting in improved resident-centered care.

